# Targeting Kinesins for Therapeutic Exploitation of Chromosomal Instability in Lung Cancer

**DOI:** 10.3390/cancers17040685

**Published:** 2025-02-18

**Authors:** Christopher Zhang, Benson Z. Wu, Kelsie L. Thu

**Affiliations:** 1 Department of Laboratory Medicine and Pathobiology, University of Toronto, Toronto, ON M5S 1A1, Canada; 2 Keenan Research Centre for Biomedical Science, St. Michael’s Hospital, Unity Health Toronto, Toronto, ON M5B 1T8, Canada

**Keywords:** kinesin, chromosomal instability, lung cancer, targeted therapy

## Abstract

New therapeutic approaches that antagonize tumour-promoting phenotypes in lung cancer are needed to improve patient outcomes. Chromosomal instability (CIN) is a hallmark of lung cancer characterized by the ongoing acquisition of genetic alterations that include the gain and loss of whole chromosomes or segments of chromosomes as well as chromosomal rearrangements during cell division. Although it provides genetic diversity that fuels tumour evolution and enables the acquisition of aggressive phenotypes like immune evasion, metastasis, and drug resistance, too much CIN can be lethal because it creates genetic imbalances that disrupt essential genes and induce severe proteotoxic and metabolic stress. As such, sustaining advantageous levels of CIN that are compatible with survival is a fine balance in cancer cells, and potentiating CIN to levels that exceed a tolerable threshold is a promising treatment strategy for inherently unstable tumours like lung cancer. Kinesins are a superfamily of motor proteins with many members having functions in mitosis that are critical for the correct segregation of chromosomes and, consequently, maintaining genomic integrity. Accordingly, inhibition of such kinesins has been shown to exacerbate CIN. Therefore, inhibiting mitotic kinesins represents a promising strategy for amplifying CIN to lethal levels in vulnerable cancer cells. In this review, we describe the concept of CIN as a therapeutic vulnerability and comprehensively summarize studies reporting the clinical and functional relevance of kinesins in lung cancer, with the goal of outlining how kinesin inhibition, or “targeting kinesins”, holds great potential as an effective strategy for treating lung cancer.

## Simple Summary

Lung cancer is a deadly malignancy and new treatments are needed to improve survival rates. Many lung tumours exhibit a feature called chromosomal instability, which is characterized by ongoing acquisition of genetic alterations that promote tumour aggression. Interestingly, chromosomal instability is a “double-edged sword”. A moderate level can support tumour growth and progression but a high level causes cell death. Therefore, chromosomal instability represents a weakness in cancer cells that could be exploited to kill lung tumours. Kinesins are a family of proteins with several members having key roles in chromosome segregation during mitosis. Because of this function, inhibiting mitotic kinesins can amplify chromosomal instability. This review summarizes the potential of targeting kinesins to elevate chromosomal instability to lethal levels as a strategy for killing chromosomally unstable lung cancers.

## 1. Introduction

### 1.1. Lung Cancer Background

Worldwide, lung cancer was responsible for nearly 2.5 million new cancer cases and over 1.8 million deaths in 2022 [1]. This indicates the immense burden lung cancer imposes on patients and families affected and the associated toll on global economies and healthcare systems. Global lung cancer incidence mirrors the prevalence of tobacco smoking in many countries [2], although up to 25% of lung cancers arise in non-smokers [3]. Despite advances in early detection due to the implementation of screening programs and translation of new treatment strategies, the 5-year survival rate for lung cancer patients remains between 10% and 20% in most countries [2]. The two major histological types of lung cancer are non-small cell lung cancer (NSCLC; ~85%) and small cell lung cancer (SCLC; ~15%) [4]. NSCLC is further subdivided into 3 subtypes with different histological and molecular features, including lung adenocarcinoma (LUAD), squamous cell carcinoma (LUSC), and large cell carcinoma, of which LUAD is the most commonly diagnosed (~40%) [5]. Lung cancer therapy is dependent on the histological subtype and stage of the tumour, as well as the presence or absence of response-predictive DNA or protein biomarkers, including oncogenic mutations and PD-L1 expression. Standard of care treatments include surgery for early-stage tumours, radiation, chemotherapy, immunotherapy, or combinations of these modalities, as well as targeted therapies that antagonize oncogenic drivers such as EGFR and KRAS inhibitors [6]. Although the therapeutic landscape has greatly expanded over the past two decades, treatments are rarely curative, and the fact remains that lung cancer patients exhibit some of the poorest survival outcomes of all cancer patients [1]. This emphasizes the need for innovative treatment strategies to improve survival rates.

### 1.2. Chromosomal Instability in Cancer

Genomic instability is a hallmark of many malignancies characterized by the ongoing acquisition of DNA alterations, including mutations, translocations, insertions, deletions, and gains and losses of chromosomal segments and/or entire chromosomes during cell division [7,8]. Several mechanisms have been identified to cause genomic instability, including compromised DNA damage response and repair pathways, chromosome segregation errors driven by defective cell cycle checkpoints, telomere shortening, and centrosome amplification, among others [9,10]. It can be broadly categorized into nucleotide instability that induces single nucleotide variants (i.e., mutations), microsatellite instability that drives expansion and/or contraction of short repeat sequences, and chromosomal instability (CIN, the type relevant for this review), which causes structural and/or numerical changes in chromosomes. Chromosomal alterations and aneuploidies induced by CIN provide genetic diversity that fuels tumour evolution and adaptation [8,10]. As a result, CIN is considered an enabling feature of cancer cells because it allows them to acquire additional malignant phenotypes like drug resistance, immune evasion, and the ability to metastasize [8,10,11]. In recognition of its multifaceted contributions to tumour progression, targeting CIN has emerged as an attractive therapeutic paradigm in oncology [9,12].

Lung cancers are among the most genomically unstable tumour types, often exhibiting high copy number alteration burdens caused by instability at the chromosomal level [13,14,15]. Thus, targeting CIN could be an effective treatment approach for lung cancer patients. Herein, we describe the concept of CIN as an actionable vulnerability in lung cancer. We then introduce the kinesin family of motor proteins and comprehensively summarize evidence implicating specific kinesins as promising targets whose inhibition could be leveraged to kill genomically unstable lung tumours. Finally, we offer our perspective on challenges that must be addressed to realize the therapeutic potential of targeting kinesins in lung and other cancers.

## 2. Exploiting Chromosomal Instability (CIN) for Cancer Therapy

### 2.1. CIN: A Vulnerability in Cancer Cells

A low to moderate level of CIN is advantageous for tumour progression, but excessive instability causes severe proteotoxic, metabolic, replication, and mitotic stress that can be fatal [10]. As a result, CIN in cancer has been described as a “double-edged sword”, requiring tumour cells to navigate a fine balance between the benefits of tumour-promoting genetic diversity and the consequences of exceeding a tolerable threshold [10,16]. However, the recurrence of CIN in cancer genomes suggests its benefits outweigh the costs, rendering tumours vulnerable to additional instability [9,10]. As such, unstable cancer cells must rely on tolerance mechanisms to preserve some level of stability to mitigate CIN’s potentially lethal consequences. Based on this premise, inhibition of such mechanisms could be leveraged to amplify instability to lethal levels. Since non-malignant cells are euploid, genomically stable, and have functional checkpoints in place to maintain genomic integrity and guard against the proliferation of abnormal cells, this treatment approach could preferentially target cancer cells. As described in Section 3, kinesins facilitate accurate chromosome segregation during mitosis, making their function critical for maintaining genome stability. Therefore, kinesins represent targets for exacerbating CIN to induce lethality in unstable cancer cells. Below, we briefly mention distinct strategies that demonstrate the concept of targeting CIN before focusing the remainder of this review on kinesins and their therapeutic potential in lung cancer.

### 2.2. Strategies for Targeting CIN in Cancer

Several approaches for exploiting CIN have been proposed, such as inhibiting proteins involved in genome maintenance or proteins that mitigate stress induced by CIN (e.g., proteotoxic and metabolic stress). Examples of the latter include potentiating CIN-associated stress with inhibitors of heat shock proteins like HSP90 or agonists of AMPK [8]. An approach illustrating the former involves abrogating the spindle assembly checkpoint (SAC) by inhibiting MPS1 (also known as TTK), the kinase that activates the SAC. Briefly, MPS1 inhibition forces mitotic progression before chromosomes are properly attached to the mitotic spindle, resulting in lethal chromosome segregation errors and mitotic catastrophe [17]. Other cell cycle kinases like PLK1, PLK4, AURKA, AURKB, and WEE1 that regulate processes critical for faithful chromosome segregation such as mitotic entry, centrosome duplication, spindle assembly, and spindle-kinetochore attachments can also be targeted to induce lethality by potentiating CIN [9,10,12]. The therapeutic efficacy of PARP inhibitors in ovarian and triple-negative breast cancer patients whose tumours harbour *BRCA1* or *BRCA2* mutations that promote CIN illustrates the potential for similar synthetic lethal strategies to be effective in the clinic [18]. Given their influence on CIN, kinesin proteins are also attractive targets in this regard.

## 3. Kinesins: Classification, Structure, and General Functions

Kinesins are a superfamily of unidirectional motor proteins that bind and move along microtubules powered by ATP hydrolysis [19,20]. To date, 45 kinesin genes have been identified in the human genome. Kinesin proteins are broadly classified into families defined based on structural similarity in their motor domains [19,20]. They can also be subdivided into three distinct groups—N-, M-, and C-type kinesins—based on the location of their motor domain. N-kinesins contain a motor domain in the N-terminal region and are plus-end-directed, travelling towards the rapidly growing plus end of microtubules. C-kinesins have a motor domain in the C-terminal region and are minus-end-directed, travelling towards the slower-growing end of microtubules anchored to the centrosome. The motor domain of M-kinesins is located in the middle of the protein, and these kinesins are non-motile.

Structurally, all kinesins contain a motor domain, a coiled-coil stalk, and a tail domain [20,21]. The highly conserved motor domain, also referred to as the head, contains binding sites for ATP and microtubules. Kinesin motor domains exhibit 30–60% homology in their amino acid sequences [21]. In contrast, the tail domains of kinesins are highly variable and dictate the binding specificity of kinesins to diverse cargo such as intracellular vesicles, organelles, or macromolecules [20,21]. In addition to direct binding mediated by the tail domain, scaffold and adapter proteins can also facilitate kinesin recognition and binding to cargo [20]. Inversely, cargo unloading is commonly mediated by Ca^2+^ signalling, Rab GTPase activity, and phosphorylation of kinesins [20]. The stalk domains of kinesins are also variable, with the coiled-coil structure enabling homo- and heterodimerization with other family members [21].

**Figure 1:**
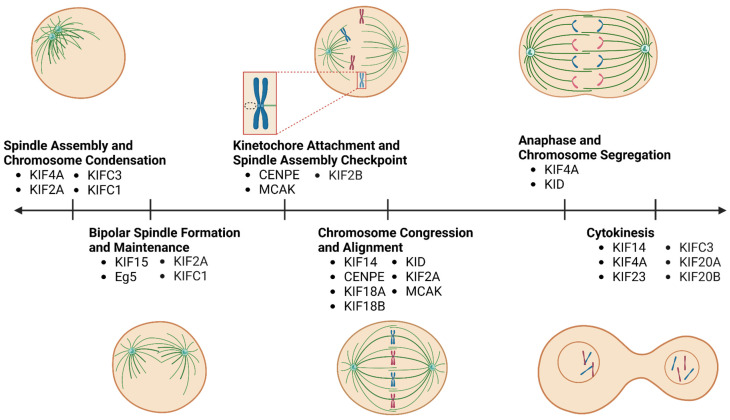
Timeline indicating the roles of kinesins in mitosis. Kinesins are involved in several critical processes during mitosis, including spindle formation, chromosome condensation, pole separation, chromosome attachment to the spindle, chromosome congression, alignment, and segregation, as well as cytokinesis. Mitotic kinesins contribute to these processes by transporting chromosomes, positioning spindle poles and microtubules, exerting push and pull forces on spindle microtubules by “walking” along them, regulating microtubule dynamics, and attaching kinetochores to K-fibres of the spindle. Note, increments between ticks of the timeline do not represent relative time intervals between subsequent stages of mitosis.

**Figure 2:**
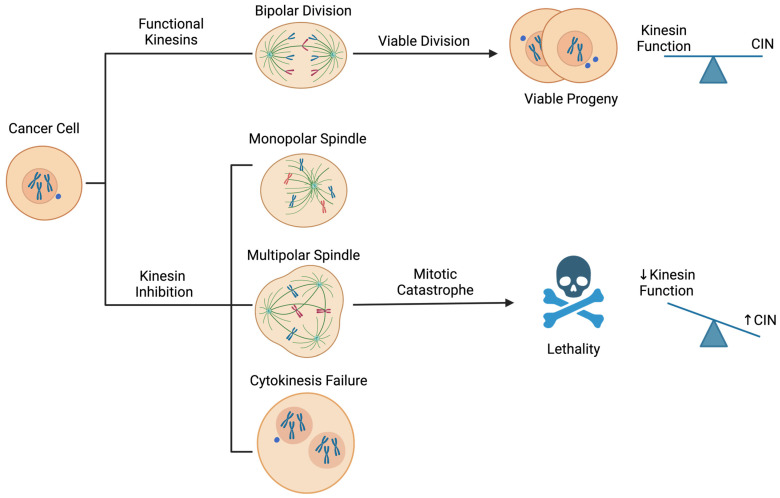
Therapeutic concept for targeting kinesins to exploit chromosomal instability (CIN) in cancer cells. Inhibition of kinesin function induces mitotic stresses, including but not limited to monopolar and multipolar spindle formation and cytokinesis failure. These abnormalities can cause severe chromosome segregation errors and aneuploidies that potentiate CIN beyond tolerable levels, leading to mitotic catastrophe and cell death immediately or upon subsequent cell divisions. Blue dots outside the nucleus indicate micronuclei.

Kinesins generally serve two functions in the cell, being involved in mitosis and/or intracellular transport. As a result, kinesins can also be classified as mitotic and non-mitotic. They have been implicated in organismal development, morphogenesis, neuronal function, and various pathologies [19,20]. Mitotic kinesins have diverse roles in mitosis, including spindle assembly, bipolar spindle formation, spindle-kinetochore attachments, chromosome congression and alignment, chromosome segregation, and cytokinesis (Figure 1, Table 1) [19,21]. They carry out these functions by leveraging their motor activity, microtubule binding, stabilization, and depolymerization abilities to (i) transport chromosomes along microtubules, (ii) exert forces on microtubules and alter their length to influence spindle assembly, positioning, and chromosome alignment, (iii) transport and organize microtubules and proteins to the spindle midzone for cytokinesis, and (iv) attach kinetochores to K-fibres, tethering chromosomes to the mitotic spindle. Given that these functions are critical for accurate chromosome segregation during cell division, many mitotic kinesins contribute to maintaining genomic fidelity during mitosis, and their loss of function enhances CIN. Accordingly, mitotic kinesins could be targeted to kill genomically unstable tumours (Figure 2). Supporting this concept, several mitotic kinesins have been reported as vulnerabilities and therapeutic targets in lung cancers with CIN, as discussed below. Although our review focuses on kinesins as targets for exacerbating CIN to lethal levels in cancer cells, it is also important to recognize that some of the therapeutic effects associated with kinesin inhibition, particularly for non-mitotic kinesins (described in Section 5), are likely attributable to their functions in intracellular transport (Table 1).

## 4. Kinesins in Lung Cancer

In this section we present the findings of studies that have identified kinesins as clinically or functionally significant in lung cancer with respect to patient characteristics and prognosis, and/or their involvement in lung tumour biology and progression. We also discuss kinesin-targeted inhibitors where relevant. Although we focus on mitotic kinesins because of their relevance for exploiting CIN, we also describe non-mitotic kinesins with reported significance in lung cancer.

### 4.1. Kinesin-3 Family: KIF14

KIF14 regulates cytokinesis and also has roles in the congression and alignment of chromosomes during mitosis [19]. KIF14 depletion causes chromosomal misalignment and cytokinesis failure, producing multinucleated cells that ultimately undergo cell death [19]. This is likely because tetraploid cells produced by cytokinesis failure contain extra centrosomes that induce multipolar cell divisions, resulting in segregation errors and aneuploidies that are often lethal immediately or upon subsequent attempts to divide (Figure 2) [8,22].

Transcriptomic analyses identified KIF14 as a prognostic gene whose high expression was correlated with shorter survival times in lung cancer patients [23]. KIF14 was also identified as part of a multigene expression signature predictive of poor overall survival in LUAD patients, further supporting its prognostic relevance [24,25]. Elevated KIF14 mRNA expression was also an indicator of poor outcomes in independent NSCLC cohorts, with higher expression observed in LUSC compared to LUAD tumours [26,27,28,29]. Consistently, Ling et al. identified that KIF14 mRNA expression was significantly higher in LUSC but not LUAD tumours compared to normal lung samples [23]. Conversely, Hung et al. used immunohistochemistry (IHC) to evaluate KIF14 protein expression in LUAD and found that it was downregulated in tumours compared to bronchial epithelial cells and that low KIF14 expression measured by qRT-PCR was associated with worse overall survival in LUAD patients [30]. These discordant results with respect to the prognostic significance of KIF14 in NSCLC could reflect differences in methods for quantifying its expression as well as clinical, molecular, and/or demographic differences in the patient populations examined. As such, additional studies using standardized protocols for measuring KIF14 expression in large independent cohorts are required to resolve them. Recently, KIF14 was identified as a putative target of miR-195-3p and miR-144-3p in LUAD models, with circ-0002727 functioning as a competing endogenous RNA that relieves miR-144-3p-mediated suppression of KIF14 expression [31,32]. Akinduro et al. also reported that KIF14 is a YAP target gene whose expression was reduced by the treatment of lung cancer cells with the YAP-TEAD inhibitor, verteporfin [33].

While most studies indicate that KIF14 behaves as an oncogene, it has been described to have both oncogenic and tumour suppressive effects in lung cancer [23,27,30,34]. Consistent with an oncogenic role, KIF14 is located within a minimal common region of DNA amplification on chromosome 1q that occurs in ~21% of lung cancers and is positively associated with tumour progression and metastasis in LUSC [26,35,36]. Like the gene expression studies described above, functional studies in lung cancer models have also shown conflicting results. One study reported that KIF14 depletion decreased cell proliferation and colony formation in H1299 cells [27], while another reported that knockdown enhanced migration and overexpression reduced migration and invasion in the C1-5 cell line [30]. This highlights the need to investigate KIF14 in additional models representing the major subtypes of NSCLC to resolve its therapeutic significance, which may be subtype-specific.

### 4.2. Kinesin-4 Family: KIF4A

KIF4A is a plus-end-directed mitotic kinesin involved in chromosome condensation, segregation during anaphase, and cytokinesis. It has also been linked to cellular response to DNA damage because of its interaction with BRCA2 [19,37]. Since it contains a DNA binding domain, KIF4A is considered a chromokinesin. KIF4A influences chromosome congression on the metaphase plate by generating polar ejection forces that counteract those from minus-end-directed dyneins at kinetochores [38]. Consistent with these functions, KIF4A depletion causes mitotic defects, including chromosome misalignment and missegregation, abnormal anaphase spindle dynamics, and aneuploidy [39]. KIF4A protein expression was detected by IHC in both NSCLC and SCLC tumours [40], and KIF4A transcript levels were identified as overexpressed in LUAD compared to non-malignant tissues [28,41]. Multiple studies found that high KIF4A expression at both the protein and RNA levels was associated with poor prognosis and tumour stage in LUAD [28,40,41,42,43,44,45]. KIF4A was also identified as a component of mitotic spindle- and microtubule-related gene expression signatures associated with poor survival of LUAD patients [46,47].

KIF4A knockdown reduced proliferation, viability, migration, and invasive properties of NSCLC cells in vitro, and their expression of epithelial-to-mesenchymal transition (EMT) markers [40,42,43,48]. Suppression of KIF4A led to reductions in sphere-forming capability, self-renewal capacity, and cell survival after radiation, while overexpression increased cancer stem cell (CSC) marker expression and cell motility in the A549 model [42], suggesting KIF4A could play a role in maintaining a stem-like phenotype in lung cancer cells. KIF4A has also been implicated in lung cancer response to chemotherapy. Two independent groups reported that KIF4A was overexpressed in cisplatin-resistant LUAD cells and promoted chemotherapy resistance [49,50]. Pan et al. identified that KIF4A interacted directly with lung resistance-related protein (LRP), a drug efflux pump, in A549 cells and proposed a putative mechanism whereby KIF4A contributes to cisplatin resistance by transporting LRP through the cytoplasm to the cell membrane [50]. A different study identified that KIF4A was upregulated upon treatment with cisplatin and that its depletion induced cell cycle arrest and enhanced cisplatin-induced cytotoxicity in H1299 cells, possibly by impairing BRCA2 and Rad51 responses to DNA damage [51]. Moreover, Zhang and colleagues found that KIF4A knockdown sensitized A549 to the cytotoxic chemotherapy, doxorubicin [48]. These findings indicate a potential role for KIF4A in mediating multidrug resistance through non-mitotic, intracellular transport functions or regulation of the DNA damage response. Collectively, they indicate KIF4A’s therapeutic potential in lung cancer and rationalize the development of KIF4A-targeted inhibitors that could be dually effective by antagonizing its transport and mitotic functions. To this end, Yan et al. recently mined gene expression and drug response data available in the Connectivity Map and identified WZ-3146 as a putative KIF4A inhibitor, reporting that it downregulated KIF4A expression and exhibited anti-cancer effects in glioma models [52]. However, no evidence was provided to show that WZ-3146 inhibited the enzymatic activity of KIF4A, indicating the need for experimental evidence to confirm this supposition.

### 4.3. Kinesin-5 Family: KIF11 (Eg5)

KIF11 encodes Eg5, or KSP, and is involved in bipolar spindle assembly, making it essential for accurate chromosome segregation [53]. It forms homotetramers consisting of two antiparallel dimers with motor domains at each end that crosslink spindle microtubules emanating from distinct centrosomes. With this configuration, its plus-end-directed motor activity enables Eg5 to generate forces that push antiparallel microtubules apart, driving centrosome separation and establishing a mitotic spindle with bipolar orientation [53]. Consistent with this function, Eg5 localizes to the centrosome and spindle poles during prophase, moving to the spindle microtubules during metaphase. Eg5 inhibition and overactivation have been tied to failed centrosome separation and premature centrosome separation, respectively, two phenotypes associated with centrosome amplification [53]. Inhibition of Eg5 causes the formation of monopolar or “monastral” spindles that activate the spindle assembly checkpoint and arrest cells in mitosis, leading to mitotic catastrophe and subsequent cell death in cancer models [53,54]. Extensive research has confirmed that Eg5 is associated with and enables cancer progression in a variety of malignancies, including colorectal cancer, glioblastoma, and NSCLC, among others, emphasizing its potential clinical utility as a prognostic marker and therapeutic target [53].

Several studies have identified KIF11/Eg5 as overexpressed at the RNA and protein levels in both NSCLC and SCLC compared to normal tissues, with high expression correlating with greater pathological stage, lymph node metastasis, and poor progression-free and overall survival rates [41,55,56,57,58,59,60,61,62,63]. High expression of Eg5 quantified by IHC was also found to predict brain metastasis following primary tumour resection in LUAD patients and to predict response to platinum chemotherapy combined with anti-mitotic agents in a retrospective NSCLC study [64,65]. Eg5 was also upregulated in *EGFR*-mutant H1975 and MDA-L-011 NSCLC lines resistant to EGFR tyrosine kinase inhibitors (TKIs) [66]. In SCLC, RNA expression of KIF11 was correlated with the proliferation marker Ki-67, an established prognostic indicator of tumour aggression [60]. At the molecular level, a study by Ling and colleagues found that the tobacco smoke carcinogen, benzo(a)pyrene, upregulated Eg5 expression in lung cancer cells [56], while hsa-miR-16-5p was reported to suppress it [67].

Genetic and pharmacologic inhibition of Eg5 arrested SCLC cells in the G2/M phase of the cell cycle and induced potent cytotoxicity in vitro, establishing Eg5 as a dependency in several SCLC cell lines [60,68]. Combined inhibition of Eg5 and BCL2L1 had an even stronger therapeutic effect in SCLC models [60]. Similarly in NSCLC cells, Eg5 depletion induced G2/M arrest, enhanced apoptosis, and reduced proliferation, migration, and invasion, while its overexpression promoted migration and angiogenesis [58,59]. Kato et al. also demonstrated that siRNA targeting KIF11 suppressed the growth of highly invasive H460SM NSCLC tumour xenografts in mice [69], and Good et al. reported that Eg5 inhibition elicited potent anti-tumour efficacy in a patient-derived xenograft model of lung cancer [70]. Furthermore, inhibition of Eg5 demonstrated efficacy in EGFR-TKI-resistant and cisplatin-resistant lung cancer cells, and combining Eg5 and BCL2L1 inhibitors effectively killed *EGFR*-mutant LUAD cells refractory to EGFR TKIs. These findings suggest that targeting Eg5 could be an effective approach for overcoming resistance to standard of care chemo- and targeted therapies in lung cancer [66,71,72].

The therapeutic potential of Eg5 is supported by its prominent upregulation in malignant compared to non-malignant cells. This provides a therapeutic window that has been validated in preclinical models, as cancer cells have shown greater sensitivity to Eg5 inhibition than normal cells [73]. Additional studies have demonstrated that Eg5 depletion exhibits greater cytotoxicity in tetraploid cancer cells compared to diploid counterparts, consistent with the idea that Eg5 can be exploited as a target in cancers with aneuploidy and/or CIN [74]. The robust anti-cancer effects of Eg5 suppression seen across diverse preclinical cancer models spurred the discovery and development of numerous Eg5 inhibitors, including but not limited to monastrol, Ispinesib, SB-743921, and Arry-520 (Filanesib). A number of these drugs have been evaluated in clinical trials [53]. Encouragingly, Eg5 inhibitors have been well tolerated and have not induced neurotoxicities commonly seen with microtubule inhibitors, but their therapeutic efficacy has been poor [75]. However, Filanesib was found to be effective in multiple myeloma when combined with the proteasome inhibitor, bortezomib, and the anti-inflammatory agent, dexamethasone [76]. Ongoing research is being conducted to develop additional Eg5 inhibitors and to understand how their clinical efficacy can be improved by discovering response predictive biomarkers, synergistic therapeutic combinations, and mechanisms of drug resistance. A recent study used a chemoinformatics approach to identify compounds capable of inhibiting Eg5, which were functionally screened in a panel of lung cancer cell lines. This revealed (-)-Cochlactone A, Phelligridin C, Sterenin E, and Cyathusal A as Eg5 inhibitors with cytotoxic activity, nominating these compounds as scaffolds for developing additional Eg5-targeted drugs with enhanced anti-tumour efficacy [59,60].

### 4.4. Kinesin-6 Family: KIF20A (MKLP2), KIF20B (MPP1), and KIF23 (MKLP1)

KIF20A encodes mitotic kinesin-like protein 2 (MKLP2), a plus-end-directed kinesin essential for cytokinesis that has also been implicated in regulating Golgi apparatus positioning and transport via interactions with the small GTPase, Rab6 [20]. Accordingly, its inhibition using antibodies blocked cytokinesis and produced binucleated cells [77]. MKLP2 accumulates in the nucleus during prophase and is found in the cytoplasm during metaphase [78]. Later in mitosis, it complexes with the chromosomal passenger complex to facilitate cleavage furrow formation [78]. Phosphorylation by PLK1 is known to regulate MKLP2’s motor activity and ability to mediate cytokinesis [19,37]. With respect to its transcriptional regulation, KIF20A has been identified as a target of Hedgehog (Hh) signalling [79,80] and of FOXM1 in response to radiation [81].

KIF20A/MKLP2 is overexpressed in many cancers, including lung cancer, raising interest in its potential as a therapeutic target [78]. Overexpression of KIF20A RNA has been reported in several studies that analyzed NSCLC and SCLC tumours and is associated with poor patient prognosis; KIF20A was also identified as a component of prognostic gene expression signatures associated with drug resistance and microtubules [28,41,46,47,82,83,84,85,86,87,88,89]. Zhao et al. demonstrated ubiquitous MKLP2 expression in LUAD tissues but absent or weak staining in matched non-malignant tissues by IHC. They also reported that high expression was associated with poor prognosis, suggesting KIF20A’s prognostic utility is consistent regardless of whether RNA or protein is assessed [41,46,82,83,84,85,86,87,88].

Multiple groups have investigated the therapeutic potential of MKLP2 in lung cancer models using RNAi-based methods. MKLP2 depletion was found to reduce cell proliferation and colony-forming capacity, enhance apoptosis, and induce G1 arrest in H1975 and A549 LUAD cells in vitro, and its knockdown inhibited the growth of A549 tumour xenografts in mice [87,88]. Similarly, Sun et al. reported that MKLP2 knockdown in A549 and PC9 cells inhibited proliferation and colony formation, induced G2/M phase arrest, and enhanced apoptosis [90]. An independent group also knocked down MKLP2 in A549 and PC9 cells and observed reduced colony formation, increased reactive oxygen species production, and enhanced sensitivity to the chemotherapeutic agent, gemcitabine [91]. Lastly, KIF20A/MKLP2 expression was upregulated after treatment with radiation, and its knockdown impaired the ability of A549 cells to survive radiotherapy [81]. These latter studies implicate MKLP2 in regulating multimodal therapeutic resistance, possibly through a FOXM1-driven signalling axis, but further research is needed to confirm this hypothesis.

Its tumour-dominant expression patterns and oncogenic functions make MKLP2 an attractive target for anti-cancer therapies. As such, numerous strategies for inhibiting MKLP2 have been identified, including natural compounds and small molecule inhibitors [78,92]. Three small molecule inhibitors have been reported: Paprotrain, Compound **9a**, and BKS0349. Paprotrain is a non-competitive inhibitor of ATP and microtubules that disrupts the mobility of the chromosomal passenger complex and cytokinesis, resulting in binucleated cells without affecting other kinesin proteins [93]. Characterization of paprotrain’s effects in another study indicated that it disrupted kinetochore-microtubule attachments, resulting in chromosome alignment errors and CIN [94], providing evidence that MKLP2 inhibition could be leveraged to potentiate instability in cancer cells. Compound **9a** is an analogue of paprotrain that exhibited enhanced inhibition of MKLP2 ATPase and microtubule-stimulating activity and demonstrated promising cytotoxicity in human cancer cell lines [92,95]. Most recently, BKS0349, another derivative of paprotrain, was described as an antagonist of MKLP2, but it has not been evaluated in cancer models [96]. Interestingly, berberine, a traditional Chinese medicine, has been proposed to inhibit LUAD tumour models in vivo by suppressing KIF20A and CCNE2 expression [97]. However, whether berberine physically interacts with MKLP2 to inhibit its activity is unknown, and experimental evidence is required to support this idea. Each of these MKLP2 compounds requires rigorous studies to determine their effects on non-malignant cells and toxicities in mice to better understand their therapeutic potential.

An early finding that KIF20A expression was largely restricted to malignant tissues, particularly pancreatic cancer, with minimal expression in most adult normal tissues spurred Imai et al. to hypothesize that peptides derived from MKLP2 could be tumour-associated antigens (TAAs) and targets for cancer immunotherapy [98]. Subsequent work led to the discovery that 3 different HLA-A2-restricted MKLP2-derived peptides could activate human cytotoxic CD8+ T cells and that such T cells could effectively kill HLA-A2+ MKLP2-expressing cancer cells in vitro [98]. Several follow-up studies provided additional evidence to support the development of vaccines to stimulate anti-tumour immunity against MKLP2-derived TAAs expressed in cancer cells, which have emerged as another mode of targeting MKLP2 [92]. As recently reviewed by Moon, multiple MKLP2 vaccines have been evaluated in phase I clinical trials, including in advanced NSCLC and SCLC patients, and have demonstrated tolerability and encouraging abilities to antagonize several tumour types [92]. Although this therapeutic strategy is independent of MKLP2’s role in cell division and genome maintenance, it is a promising approach for treating lung cancers with high MKLP2 expression. Ongoing trials will determine its clinical utility.

KIF20B, also known as MPP1, is a plus-end-directed kinesin that localizes to the spindle midbody to regulate abscission, and its knockdown leads to cytokinesis failure and multinucleation of dividing cells [99]. KIF20B was identified as overexpressed at the mRNA level in the TCGA’s cohort of LUAD tumours compared to normal tissue controls, and higher expression was correlated with advanced stage and worse survival [100]. Knockdown of MPP1 reduced migration and survival and increased apoptosis in A549 cells, which was associated with elevated p53 and Bax expression and downregulation of BCL2 [100]. Although no clinical-grade inhibitors are available, a screen for MPP1 inhibitors revealed that depsidones, which are natural compounds derived from lichen, inhibited MPP1 [101]. The marine natural product, adociasulfate-2 (AS-2), was also identified as a non-specific kinesin antagonist capable of inhibiting multiple kinesins, including MPP1 [102]. These chemical probes may prove useful for informing the future development of potent and selective MPP1 inhibitors.

KIF23, also known as mitotic kinesin-like protein 1 (MKLP1), is part of the centralspindlin complex, which bundles microtubules to regulate spindle midbody formation and cytokinesis [19,37]. Loss of MKLP1 function causes cytokinesis failure and the production of binucleated and multinucleated cells [19,37]. Numerous studies have demonstrated that KIF23 mRNA and/or protein is overexpressed in NSCLC and SCLC tumours, which are associated with poor prognosis of patients, specifically greater tumour stage, and worse overall and recurrence-free survival [103,104,105,106,107,108,109,110]. One study demonstrated the absence of MKLP1 staining in non-malignant lung tissues [104]. The activity of MKLP1 is regulated through phosphorylation by multiple mitotic kinases, including AURKB, CDK1, and PLK1 [19,37]. More recently, YAP was shown to regulate KIF23 at the transcriptional level, as YAP inhibition with verteporfin or siRNA reduced KIF23/MKLP1 RNA and protein expression [33,111].

At the phenotypic level, Iltzsche et al. discovered that MKLP1 was required for the development of lung tumours in genetically engineered mouse models of NSCLC driven by the loss of p53 and KRAS activation [112]. They also showed that MKLP1 depletion with shRNA inhibited proliferation and induced apoptosis in 4 lung cancer cell lines and impaired lung tumour growth in vivo, which was associated with abnormalities including giant multinucleated cells [112]. An independent group showed that MKLP1 depletion also reduced cell proliferation in H2122 LUAD cells [104,112]. Although no specific MKLP1 inhibitors have been reported, the natural marine product AS-2 was found to inhibit MKLP1 [102], indicating its amenability to pharmacologic inhibition. Based on the evidence in preclinical models of lung and other cancers, MKLP1 represents an attractive therapeutic target warranting the development of specific inhibitors. Interestingly, kinesin-6 family members have a unique 8–10 kDa sequence in their motor domain that may provide an opportunity to develop kinesin-selective inhibitors [19].

### 4.5. Kinesin-7 Family: KIF10 (CENPE)

Centromere-associated protein-E (CENPE), encoded by KIF10, is a plus-end-directed kinesin critical for faithful chromosome segregation during mitosis because of its roles in attaching kinetochores to the spindle and chromosome alignment, as well as SAC regulation [113]. CENPE forms homotetramers that localize to the kinetochores from nuclear envelope breakdown to anaphase [114]. Accordingly, CENPE loss of function increases chromosome misalignment, mitotic arrest, aberrant spindle positioning, CIN, and mitotic catastrophe in cancer, fibroblast, and liver regeneration models [113,115]. Given its involvement in suppressing CIN, CENPE has emerged as a promising therapeutic target in several malignancies [113].

Numerous independent analyses of gene expression databases, clinical tumour samples, and in vitro cell lines have revealed that KIF10/CENPE is overexpressed in both LUAD and LUSC forms of NSCLC [82,116,117,118,119,120]. Upregulation of KIF10 RNA is associated with worse patient prognosis in LUAD and LUSC, although the association may be weaker in LUSC [116,117]. While all tumour grades have been shown to express elevated levels of KIF10 compared to non-malignant tissues, multiple reports indicate that KIF10 RNA expression is significantly higher in stage II, III, and IV compared to stage I tumours and associated with worse patient outcomes [118,119]. Transcriptomic analyses of clinical NSCLC samples identified KIF10 as a hub gene implicated in various cellular processes, including cell division, cell cycle, DNA replication, angiogenesis, and cell migration [121]. Another correlative study discovered that somatic mutations in the *KIF10* gene were associated with distant recurrence in patients, leading the authors to speculate that genetic disruption of KIF10 drives lung cancer progression by enhancing CIN [122]. *KIF10* mutations were also identified in tumour tissues of patients with sporadic lung cancers, as well as in the constitutional DNA of multiple members of a family with suspected familial lung cancer, nominating germline *KIF10* mutations as putative lung cancer risk factors [123].

While most studies of CENPE in lung cancer have been correlative, some have studied its loss of function using genetic or pharmacological means, revealing that CENPE inhibition reduces proliferation and colony-forming capacity and arrests cells in G2/M in multiple lung cancer cell lines (A549, PC9, H460, H522) [117,118,120]. The study by Shan et al. additionally discovered a putative regulatory relationship between CENPE and the FOXM1 transcription factor, indicating that FOXM1 binds to the KIF10 gene promoter to control its expression [118]. Since FOXM1 is a downstream target of several oncogenic pathways, including PI3K/Akt, Ras/ERK, and JNK/p38 MAPK, it is possible that CENPE is induced to support mitotic fidelity in rapidly proliferating cells [118,124].

Several inhibitors of CENPE exist and were described in a recent review [113]. Of these, GSK923295 has been the most studied in preclinical models, and a first-in-human phase I clinical trial revealed it had an acceptable toxicity profile and resulted in stable disease in one-third of previously treated patients with solid tumours [125,126]. GSK923295 is a potent and specific allosteric inhibitor of CENPE that has been shown to inhibit cell proliferation in vitro and tumour growth in vivo in lung cancer and several other malignancies [113,127,128]. However, like many anti-mitotic drugs, CENPE inhibitors have had limited clinical efficacy as monotherapies, which may be attributable to “mitotic slippage” of cancer cells that experience prolonged mitotic arrest in response to such drugs. Mitotic slippage is characterized by cells exiting mitosis before sufficient apoptotic signals accumulate to drive cell death, allowing them to continue dividing [129]. This finding has prompted the exploration of combinations to bolster the therapeutic effects of CENPE inhibitors like GSK923295.

In lung cancer models specifically, CENPE inhibition has shown promising synergistic effects when combined with Navitoclax, a BH3-mimetic that inhibits anti-apoptotic proteins, as well as MEK/ERK inhibitors [120,130,131]. Both combinations yielded enhanced anti-tumour effects compared with monotherapy and abrogated the post-mitotic survival of cells that underwent mitotic slippage in A549, H460, H522, and Lewis lung carcinoma cells [117,120]. Interestingly, CENPE inhibition with GSK923295 was recently described to synergize with PD-L1-targeted immunotherapy in NSCLC tumour models, which was attributed to CENPE inhibitor-mediated upregulation of PD-L1 [117]. Moreover, CENPE was identified as part of a 3-gene prognostic signature whose high expression correlated with worse survival and greater T cell infiltration in *EGFR*-mutant LUAD patients [132]. This finding warrants validation studies and additional investigations to determine whether CENPE expression has value for predicting immunotherapy response. In line with mitotic kinesins being strategic targets for amplifying CIN to lethal levels, Tucker et al. recently demonstrated that CENPE inhibition enhanced CIN and synergized with microtubule-disrupting agents in breast cancer [133]. Additionally, a study by Yoshizawa et al. found that the anti-proliferative effects of GSK923295 were greater in tetraploid compared to diploid HAP1 leukemia cells, suggesting that CENPE inhibitor cytotoxicity could be restricted to aneuploid tumours or cancers with CIN [134]. However, combinatorial therapeutic approaches will likely be required to achieve maximal therapeutic benefit with CENPE inhibitors.

### 4.6. Kinesin-8 Family: KIF18A, KIF18B

KIF18A functions as both a plus-end-directed motor protein and a microtubule depolymerase [135]. It localizes to the plus end of kinetochore microtubules and regulates chromosome alignment during mitosis by influencing kinetochore-microtubule dynamics in prometaphase through metaphase [19,135]. KIF18A loss of function causes a failure of chromosome alignment, hyperstable microtubules, and a loss of spindle tension that activates the SAC [19]. While KIF18A expression is low in non-malignant tissues, elevated KIF18A expression has been detected at both RNA and protein levels in NSCLC tissues, consistent with reports for several other human cancers [136,137,138,139,140]. High KIF18A RNA and protein expression in NSCLC has been associated with higher tumour stage, tumour differentiation, lymph node metastasis, and tumour mutation burden [136,138,139,140]. Concordantly, multiple studies have identified KIF18A as a prognostic marker in LUAD, as elevated KIF18A expression was correlated with reduced overall survival, disease-free survival, and recurrence-free survival in LUAD but not LUSC patients [136,137,139]. KIF18A was also reported as a putative serum biomarker that could predict patients with asbestosis who have a high risk of developing lung cancer [141].

Depletion of KIF18A has been shown to induce excessive CIN and death of genomically unstable cancer cells [142]. Independent functional studies have shown that KIF18A depletion impairs proliferation, migration, and invasion; increases apoptosis; induces cell cycle arrest in the A549 and H1975 LUAD models; and also inhibits tumour growth and metastasis in in vivo tumour models [136,137,140]. A flurry of recent studies has implicated KIF18A as a tractable therapeutic vulnerability specifically in cancers with aneuploidy, CIN, weakened APC/C activity, and SAC persistence [142,143,144,145,146]. Supporting KIF18A’s therapeutic promise in genomically unstable cancers like triple-negative breast cancer and high-grade serous ovarian cancers, multiple KIF18A inhibitors have been developed and were recently reviewed by Chen and colleagues [147]. Based on encouraging preclinical studies demonstrating selective cytotoxicity in models with aneuploidy and CIN compared to diploid and genomically stable counterparts, several KIF18A inhibitors have progressed to evaluation in clinical trials [147]. Notably, one inhibitor, GSC000190, appears to exhibit anti-cancer efficacy in tumours resistant to cisplatin and olaparib, highlighting the potential utility of targeting KIF18A in the context of therapy resistance, which is often associated with CIN [148]. Given these exciting discoveries, the oncology field ardently awaits the results of ongoing clinical trials.

KIF18A’s kinesin-8 family member, KIF18B, is also an N-type plus-end-directed microtubule depolymerase that localizes to the plus-ends of microtubules to regulate astral microtubule length and chromosome alignment [149]. It complexes with MCAK (KIF2C) to control microtubule depolymerization [149]. KIF18B has been identified as a differentially expressed, upregulated gene in both LUAD and LUSC tumours in the TCGA database based on transcriptomic analyses [28,41,44,150]. Additionally, it was identified as upregulated in LUAD tumours compared to normal tissues using IHC, Western blotting, and qPCR approaches [150,151,152]. High KIF18B RNA levels were reported to correlate with worse overall survival, but only in LUAD patients [28,41,44,150,151]. Additional correlative analyses in LUAD models revealed that cell lines with higher basal proliferation rates had higher expression of KIF18B compared to those with lower proliferation rates [44]. In the same study, gene set enrichment analyses of TCGA LUAD tumours with high versus low KIF18B mRNA expression suggested KIF18B-high tumours were enriched for gene expression signatures indicative of mTORC1 signalling, G2/M checkpoint, MYC, E2F, and mitotic spindle regulation pathways, consistent with KIF18B’s roles in mitosis and its correlation with a proliferative capacity [44]. Functionally, the knockdown of KIF18B reduced proliferation, migration, and invasion of A549 cells, which was associated with reductions in the expression of Rac1-GTP, phospho-AKT, and phospho-mTOR [151], suggesting it may also be therapeutically relevant in lung cancer.

### 4.7. Kinesin-10 Family: KIF22 (KID)

KIF22, or kinesin-like DNA-binding protein (KID), is a monomeric chromokinesin capable of binding to microtubules through both its motor and tail domains and to DNA via its tail domain [19]. It localizes to spindle microtubules and chromosomes to regulate chromosome congression and alignment by generating polar ejection forces. As such, KID loss of function leads to mitotic defects, including chromosome misalignment, shortened spindles, and anaphase delay [19]. KID also regulates chromosome compaction during anaphase, and its depletion increases the prevalence of lagging chromosomes and multinucleated cells [153]. KIF22 was identified as a significantly upregulated gene in LUAD tumours at the RNA level but was not associated with patient prognosis [28].

Pike et al. explored the functional relevance of KID in lung cancer. In A549 cells, KID depletion reduced EGF-dependent proliferation, which was associated with a reduction in ERK phosphorylation, suggesting KID’s involvement in regulating EGFR signalling [154]. EGF was also shown to stimulate the binding of KID to the coxsackie and adenovirus receptor (CAR), implicating CAR in regulating EGFR signalling through its interaction with KID. Moreover, KID was identified to influence cytoskeletal dynamics in an EGF-dependent manner by stabilizing microtubules, which contributed to the retention of EGFR at the plasma membrane [154]. Therefore, although further studies are required to understand the generalizability of these findings, KID inhibition could potentially suppress lung cancer growth by disrupting its contributions to maintaining genome stability and EGFR signalling.

### 4.8. Kinesin-12 Family: KIF15

KIF15 is a homotetrameric kinesin involved in spindle elongation and maintenance of bipolarity [19,155]. It was reported to regulate the length of K-fibres and to form a complex with TPX2 that crosslinks and slides microtubules apart to separate centrosomes during spindle assembly [155]. Interestingly, KIF15 was identified as non-essential for bipolar spindle assembly when Eg5 is active but essential upon loss of Eg5 activity [155], indicating that KIF15 compensates for Eg5 when necessary. KIF15 RNA and protein expression were found to be higher in LUAD and LUSC tumour tissues compared to normal tissues based on mining gene expression databases, IHC, and immunoblotting [28,155,156,157,158]. High RNA and protein expression of KIF15 correlated with worse overall survival of LUAD but not LUSC patients [28,41,156,157], and KIF15 was identified as a component of metabolism-, microtubule-, and mitotic spindle-associated gene expression signatures that correlated with poor survival in LUAD [46,47,159]. High KIF15 protein levels were also found to correlate with advanced tumour stage [157].

Multiple studies have indicated KIF15’s functional relevance in lung cancer. Two independent groups found that KIF15 depletion inhibited proliferation, migration, and invasion of lung cancer cells and that these anti-cancer effects were only seen in LUAD (A549, H1299) and large-cell carcinoma (H460) models with no effect in LUSC (H226) cells [156,157]. KIF15 knockdown in NSCLC cell lines also increased apoptosis and induced G1 arrest, which was associated with reduced Cyclin D1 and increased p27 expression [156,157]. Furthermore, the growth of H460 lung tumour xenografts expressing KIF15-targeted shRNA was significantly impaired in mice, and stunted tumour growth was associated with the downregulation of components of the Raf-Mek-ERK signalling pathway, including phospho-ERK, ATF2, phospho-MEK, and phospho-c-Raf [156]. Taken together, these studies suggest KIF15 has therapeutic potential in lung cancer, particularly LUAD, due to its positive regulation of oncogenic signalling pathways. Given its functions in mitosis, KIF15 inhibition is likely to influence genomic stability; however, the effects of its inhibition on CIN and mitotic phenotypes remain to be determined in lung cancer cells. The ability of KIF15 to compensate for loss of Eg5 function led Rath and Kozielski to hypothesize that KIF15 can confer resistance to Eg5 inhibitors, which was later demonstrated in multiple studies [160,161]. Accordingly, KIF15 inhibitors have demonstrated synergy with Eg5 inhibition [162,163], rationalizing further development of KIF15-targeted agents and studies to characterize this therapeutic combination. While multiple KIF15-targeting tool compounds have been reported (e.g., GW108X, Munesib-1, Fift-IN, dihydropyrazole and dihydropyrrole derivatives), none have progressed beyond preclinical stages of development [163,164,165].

### 4.9. Kinesin-13 Family: KIF2A, KIF2B, KIF2C, and KIF24

KIF2A regulates microtubule dynamics to influence spindle assembly and chromosome alignment through its microtubule depolymerizing activity [19]. It localizes to spindle microtubules and spindle poles during mitosis. Cells lacking KIF2A form monopolar spindles, indicating its importance for bipolar spindle assembly [19]. KIF2A is overexpressed in NSCLC cell lines compared to non-malignant lines [166,167] and protein expression is upregulated in NSCLC tumours relative to non-malignant tissues [168,169]. KIF2A expression is also clinically relevant, being associated with multiple prognostic factors in lung cancer patients. For instance, high KIF2A expression was correlated with tumour stage and lymph node metastasis in a cohort of LUAD tumours [168]. A larger study of NSCLC patients found that high KIF2A protein expression was associated with increased pathological tumour grade and size, the presence of lymph node metastasis, and worse disease-free and overall survival [169].

Functional studies in lung cancer models have implicated KIF2A in regulating multiple oncogenic phenotypes. KIF2A knockdown decreased cell viability, increased apoptosis, decreased invasive properties, and reduced expression of EMT markers in A549, H1975, and H1299 LUAD cells [166,170]. Concordantly, KIF2A overexpression promoted cell proliferation, migration, and invasion in NSCLC cells in vitro [167,169]. KIF2A knockdown was also reported to reduce stem-like features of LUAD cell lines, including sphere-forming ability and the proportion of CD133+ cells [166,167]. KIF2A was also shown to influence lung cancer response to chemotherapy, as knockdown sensitized A549, H1975, and H1299 cells to cisplatin and paclitaxel [166,169], while overexpression conferred resistance to cisplatin [167]. Moreover, KIF2A influences the Notch, Wnt, PI3K-Akt, and VEGF signalling pathways [166]; its knockdown was found to reduce the expression of PI3K, phospho-AKT, and VEGF [167], and its overexpression increased Wnt1, GSK-3, and β-catenin levels [170]. Expression of KIF2A in NSCLC cells can be regulated by multiple non-coding RNA axes, including circ_0010235-mediated regulation of miR-338-3p and circ_SATB-mediated regulation of miR-760, as well as miR-451a, as each of these microRNAs targets KIF2A to reduce its expression [171,172,173]. Recruitment of KIF2A to spindle microtubules and its depolymerase activity is further regulated in a cell-cycle-specific manner by multiple kinases, including PLK1, AURKA, and AURKB [19].

KIF2C, also known as mitotic centromere-associated kinesin (MCAK), also depolymerizes microtubules to regulate kinetochore-microtubule attachments as well as chromosome congression and alignment [19]. Depleting MCAK in non-malignant cells induces chromosome missegregation and CIN, demonstrating its role in maintaining genomic integrity [19]. KIF2C has also been implicated in DNA repair, cellular senescence, and immune modulation, although the mechanisms underlying these putative functions are not well understood [174]. Multiple independent studies of NSCLC have demonstrated that lung tumours express higher levels of KIF2C mRNA than normal lung tissues, and that high expression is associated with a worse prognosis of NSCLC patients [28,41,175,176,177]. Specifically, high KIF2C expression correlated with greater tumour stage, lymph node metastasis, disease recurrence, and worse overall survival [174,176,177,178]. Moreover, KIF2C was a component gene of multiple mRNA signatures associated with poor prognosis in lung cancer patients [23,179,180].

In cultured H1299 and A549 LUAD and H226 LUSC models, MCAK silencing inhibited cell proliferation, promoted apoptosis, and impaired migration, invasion, and EMT [175,176,177]. In vivo, knockdown of MCAK reduced the growth of LA-4 tumour xenografts [177], and an independent group found that the reduction in lung tumour size associated with MCAK knockdown was associated with increased E-cadherin and suppressed vimentin expression [177], implicating MCAK in EMT. MCAK has also been shown to promote resistance to microtubule-disrupting and DNA-damaging chemotherapies in lung and other cancer models [174]. At the cellular level, KIF2C overexpression resulted in increased expression of β-catenin, phosphorylated GSK-3β, and phosphorylated AKT, suggesting MCAK influences Wnt/β-catenin signalling [175]. Its own expression can be regulated by miR-186-3p, which is often downregulated in lung cancer cells. Transfection with miR-186-3p phenocopied MCAK knockdown in LUAD cells, slowing proliferation, increasing apoptosis, and reducing expression of several Wnt/β-catenin pathway components [175]. Given that it supports multiple malignant phenotypes and chromosome segregation, MCAK is an attractive therapeutic target that could be inhibited to potentiate CIN to lethal levels. In support of this idea, MCAK inhibition has been shown to induce aneuploidy and reduce the viability of genomically unstable cancers like triple-negative breast cancer [181], warranting additional research to develop MCAK-targeted drugs.

The other two members of the kinesin-13 family, KIF2B and KIF24, which are also microtubule depolymerases, have been less studied in the context of lung cancer. Laucius et al. found that KIF2B influences CIN by reducing chromosome segregation defects in a genetically engineered mouse model of Kras-driven lung cancer [182]. This finding is consistent with the established role of KIF2B in maintaining genomic integrity by regulating and correcting kinetochore-microtubule attachments [19,183]. KIF24’s involvement in mediating microtubule dynamics has been shown to regulate cilia formation [184]. Mutations in *KIF24* are associated with NSCLC metastasis [185], but functional studies are required to confirm whether this can be explained by its regulation of cilia assembly.

### 4.10. Kinesin-14A Family: KIFC1

KIFC1, or HSET, the lone kinesin-14A member, is a minus-end-directed homodimeric kinesin whose functions include spindle formation and pole focusing, as well as vesicle, organelle, and double-stranded DNA transport [19,186]. KIFC1 is highly expressed in many cancers at both the RNA and protein levels, including breast, ovarian, bladder, prostate, and kidney tumours, among others [186,187,188]. In lung cancer, both RNA and protein expression of KIFC1 is elevated in LUAD, LUSC, and SCLC tumours compared to healthy lung tissues, which may be attributable to loss of regulatory DNA methylation (i.e., hypomethylation) at the *KIFC1* genomic locus [41,189,190,191]. Elevated KIFC1 expression in NSCLC is correlated with advanced TNM stage, lymph node metastasis, smoking history, and poor patient prognosis, specifically worse progression-free and overall survival [189,190,191,192]. Moreover, overexpression of KIFC1 was found to be enriched in tumours with *TP53* mutations [193]. KIFC1 was identified as a component gene in an expression signature predictive of NSCLC metastasis to the brain [194], and its expression was elevated in the serum of lung cancer patients compared to healthy individuals [190], indicating its potential utility as a prognostic biomarker.

Functionally, KIFC1 depletion is associated with various anti-cancer effects, including suppressed proliferation, colony formation, cell growth, invasion, and migration, while its overexpression enhanced malignant phenotypes in H1299, PC9, A549, and SPC-A1 lung cancer cells [189,191,192]. Notably, it has been described as non-essential in non-malignant cells, providing a therapeutic window for targeting KIFC1 [195,196]. Silencing of KIFC1 has also been shown to impair EMT, evident by expression changes in E-cadherin, N-cadherin, and Vimentin in LUAD cells, by regulating the TGF-β/SMAD signalling pathway [192,197]. Gene set enrichment analyses comparing TCGA lung tumours with high and low KIFC1 mRNA expression suggested KIFC1 involvement in the cell cycle, DNA replication, and TP53 signalling pathways [193].

Using a CRISPR/Cas9 screen, we recently discovered KIFC1 as a vulnerability specifically in LUAD cells with centrosome amplification (CA), a malignancy-associated phenotype known to drive CIN [189,191,192]. The presence of extra centrosomes during mitosis leads to multipolar mitotic spindles that are prone to causing major chromosome segregation errors, lethal aneuploidies, and mitotic catastrophes if left unmanaged [22]. Our assessment of KIFC1 dependency across 6 LUAD models confirmed that KIFC1 depletion only reduced the viability of cell lines with high basal levels of CA and KIFC1 expression (H1299, H1975, H2030), nominating these features as putative biomarkers for predicting sensitivity to KIFC1 inhibition [189,191,192]. Mechanistically, loss of KIFC1 function in vulnerable models was associated with an inability to effectively group extra centrosomes into mitotic spindles with a bipolar orientation, attributable to KIFC1’s role in centrosome clustering and pole focusing [189,191,192].

Due to its promotion of several oncogenic phenotypes and the dependence of cancer cells on KIFC1 to avoid the deleterious effects of CA-associated multipolar mitotic spindles, KIFC1 is a promising target in cancers in which CA is prevalent [22,191]. Several compounds with the ability to inhibit KIFC1 have been described and were recently summarized and computationally evaluated in a comprehensive review [188]. Briefly, these compounds aim to antagonize the centrosome clustering function of KIFC1 to induce severe chromosome segregation errors that lead to intolerable CIN and mitotic catastrophe. However, it is important to note that they may also exert therapeutic effects by blocking KIFC1-mediated intracellular transport of macromolecules and organelles. Despite their utility as tool compounds, none of these agents have progressed to clinical studies due to a lack of potency, selectivity for KIFC1, poor pharmacological profiles, or a combination of these limitations. Thus, continued efforts are required to develop effective strategies for targeting KIFC1, requiring advances in medicinal chemistry and improved knowledge of KIFC1 structure to exploit its therapeutic potential [188].

### 4.11. Kinesin-14B Family: KIFC3

KIFC3 is a tetrameric kinesin that contributes to multiple mitotic processes, including centrosome cohesion, spindle assembly, and cytokinesis, as well as the transport of intracellular cargo [20,198,199]. It was recently reported to localize to the cytoplasm and nucleus and was identified as overexpressed and positively correlated with tumour size, stage, lymph node metastasis, and worse overall survival and poor prognosis in NSCLC patients [200]. Functionally, KIFC3 knockdown reduced proliferation, colony formation, migration, invasion, and expression of phospho-AKT, Cyclin D1, CDK4, CDK6, RhoA, and RhoC in A549 and H1299 cells, while its overexpression enhanced these phenotypes [200]. Overexpression of KIFC3 also enhanced subcutaneous tumour growth and metastasis to the lungs in xenograft models of A549 and H1299 [200,201]. As such, KIFC3 appears to promote multiple phenotypes associated with lung cancer growth and progression.

## 5. Non-Mitotic Kinesins Implicated in Lung Cancer

A number of non-mitotic kinesins have been described as prognostically or functionally relevant in lung cancer, with putative roles in regulating malignant phenotypes and oncogenic signalling pathways. Although the anti-cancer effects of targeting these kinesins are likely driven by cargo transport and other cellular functions rather than by influencing CIN, here we briefly describe evidence of their significance in lung cancer.

The functions of KIF26B (kinesin-11 family member) include regulation of cell adhesion, migration, and polarity [202,203]. A study in NSCLC found that KIF26B expression was higher in tumours compared to normal tissues and was associated with poor overall survival of patients [204]. KIF26B silencing in H1299 and H520 cell lines slowed proliferation, impaired invasion, induced cell cycle arrest in vitro, and suppressed the growth of H520 tumour xenografts in mice [204]. Knockdown also enhanced the sensitivity of lung cancer cells to cisplatin chemotherapy [204]. Furthermore, KIF26B silencing led to the downregulation of vimentin and N-cadherin, upregulation of E-cadherin, and diminished Wnt/β-catenin pathway activity, implicating KIF26B as a positive regulator of EMT and Wnt/β-catenin signalling in lung cancer cells [204]. These findings are consistent with an oncogenic role for KIF26B in NSCLC, but this remains to be corroborated in additional models.

KIF7 is a member of the kinesin-4 family and a microtubule-associated protein involved in Hedgehog signalling and cilia formation through its regulation of microtubule dynamics [205,206]. Hu et al. found that the HPV-associated oncoprotein, E6, reduced the expression of KIF7, which was associated with the downregulation of the tumour suppressor LKB1 in H1299 cells [207]. In murine respiratory epithelial cells, KIF7 was shown to negatively regulate microtubule stability, S phase entry, mitotic exit, and cell proliferation [208]. KIF7 loss of function was associated with an increase in Hedgehog effector proteins, GLI1 and GLI2 [208]. Unlike other kinesins, these findings are consistent with KIF7 behaving as a tumour suppressor in lung cancer. Its regulation of the cell cycle, particularly mitotic exit, suggests it may influence genomic integrity during mitosis, but functional experiments are required to support this idea.

KIF21B is another kinesin-4 family member that regulates cytoskeletal transport, neuronal morphology, and microtubule dynamics by inhibiting the growth of microtubules through its tail domain [209,210]. Sun et al. investigated the clinical and functional relevance of KIF21B in NSCLC [211]. IHC staining for KIF21B revealed it was localized primarily to the cytoplasm and was highly overexpressed in tumours compared to normal lung tissues. Moreover, high KIF21B expression was positively correlated with TNM stage and worse prognosis. In functional experiments, KIF21B silencing selectively reduced the proliferation, colony formation, migration, and invasion capacity of two LUAD cell lines (H1299 and A549) compared to non-malignant lung cells (BEAS-2B). Knockdown also increased apoptosis in lung cancer models, which was associated with reduced expression of BCL2, reduced phosphorylation of Akt, and decreased Cyclin D1 expression specifically in malignant models [211]. Finally, they found that KIF21B-targeted shRNA significantly impaired the growth of H1299 lung tumour xenografts in nude mice [211]. The finding that KIF21B promotes multiple oncogenic phenotypes suggests that KIF21B may be a promising therapeutic target that warrants further exploration in NSCLC.

The kinesin-1 family includes the neuron-specific KIF5A and ubiquitously expressed KIF5B, both of which are involved in the axonal transport of various cargoes including organelles, neurofilaments, proteins, and RNA [212]. KIF5A was identified as a putative serum biomarker that could predict lung cancer risk in patients with asbestosis and has also been associated with resistance to docetaxel in LUAD models, consistent with its role in mediating drug resistance in other cancer types [141,213]. KIF5B is a common translocation partner of the oncogenes ALK, RET, and MET in NSCLC tumours [214,215,216,217]. These oncogenic rearrangements drive constitutive activation of the receptor tyrosine kinases involved because the 5′ end of the fusions contains a portion of KIF5B’s coiled-coil domain, which enables dimerization that mimics activation normally induced by ligand binding to the wild-type receptor. As a result, fusions stimulate receptor activation and downstream tumour-promoting functions [218].

Members of the kinesin-3 family, KIF13A and KIF13B, are also involved in genetic rearrangements, with KIF13A-RET, KIF13A-ALK, and KIF13B-NRG1 being identified in LUAD or not otherwise specified lung cancer patients [219,220,221]. KIF13A regulates recycling endosomes and cargo transport, while KIF13B is involved in endocytosis [222,223]. KIF13B has also been shown to contribute to angiogenesis by transporting VEGFR2 along microtubules to the surface of endothelial cells to stimulate angiogenic signalling [224]. A peptide designed to block the interaction between VEGFR2 and KIF13B inhibited VEGF-stimulated endothelial cell migration and the growth of human H460 lung tumour xenografts in mice [224], offering a putative anti-angiogenic strategy.

The single nucleotide polymorphism rs1555195 in the gene encoding another kinesin-3 family member, KIF16B, was associated with reduced expression of KIF16B mRNA in lung tissues and cells in the blood, as well as poor survival of NSCLC patients [225]. KIF16B is involved in endosomal transport, as well as recycling and degradation of receptors like EGFR [226]. KIF16B expression was also implicated in the growth of pre-metastatic tumours in human models of the lung-to-brain metastasis, and low mRNA expression of KIF16B was associated with poor disease-free survival in LUAD patients [227], indicating its prognostic significance in lung cancer. Similarly, KIF1C, a kinesin-3 family member that regulates the transport of mRNA and Golgi-derived vesicles to the endoplasmic reticulum [228,229], was reported as a component of a 3-gene mRNA expression signature that could predict brain metastasis in NSCLC patients, which was validated at the protein level using IHC [194].

The kinesin-2 family member, KIF3A, plays an important role in the formation and function of cilia by regulating intraflagellar transport [54,230]. KIF3A forms a heterodimer with KIF3B that functions as a plus-end-directed motor for the anterograde transport of organelles [231]. KIF3A was also found to be required for Hedgehog signalling downstream of Patch1 [232]. In lung cancer, KIF3A was identified as a fusion partner of ALK in a LUAD patient [220]. Yang et al. proposed KIF3A as a tumour suppressor in NSCLC since its knockdown in H520 cells enhanced proliferation, invasion, and migration and inhibited apoptosis [233]. Protein expression of KIF3A was significantly lower in NSCLC compared to adjacent normal tissue and lower in LUAD than in LUSC tumours; low expression was correlated with higher TNM stage in LUAD and lymph node metastasis in LUSC, revealing KIF3A as a putative prognostic factor in NSCLC [233]. Another group confirmed KIF3A’s tumour suppressive function in the human LUSC model SW900 and additionally discovered that knockdown led to hyperactivation of Wnt/β-catenin signalling, which was associated with increased proliferation, spheroid formation, migration, and expression of stemness markers in vitro as well as enhanced growth of the human LUSC tumour model SW900 in mice [234]. Kim et al. further showed that KIF3A formed a complex with β-arrestin to inhibit Wnt/β-catenin signalling, and IHC revealed that low KIF3A expression was correlated with higher β-catenin expression and poor prognosis of NSCLC patients [234]. In contrast, KIF3A was required for the development of SCLC tumours in genetically engineered mouse models driven by conditional deletion of *Trp53* and *Rb1* in the airways [235], suggesting its effects on lung tumorigenesis are lineage-specific.

Another kinesin-2 member, KIF3C, which regulates microtubule dynamics, axon growth, and regeneration, was recently described as a putative oncogene in NSCLC [236,237]. Liu et al. found that KIF3C RNA and protein expression was significantly higher in NSCLC compared to non-malignant tissues and positively correlated with TNM stage, worse overall survival, shorter progression-free survival, and shorter post-progression survival of NSCLC patients [28,236]. In functional experiments, KIF3C knockdown suppressed proliferation, migration, and invasion of A549 cells, while overexpression enhanced these phenotypes and lung metastasis in vivo in the H226 model [236]. Furthermore, both miR-150-5p and miR-186-3p were identified as negative regulators of KIF3C expression [236]. These functions are consistent with an oncogenic role for KIF3C in lung cancer, which requires validation in additional models.

## 6. Outlook and Perspective

Mitotic kinesins regulate several processes critical for faithful chromosome segregation, bestowing them with integral roles in maintaining genomic integrity. As a result, their loss of function potentiates CIN and induces severe aneuploidies, highlighting the potential to harness kinesin inhibition to induce synthetic lethality in genomically unstable cancers like lung cancer. Notably, the therapeutic potential of some kinesins can also be attributed to their regulation of intracellular transport, oncogenic signalling pathways, and other hallmarks of cancer that drive tumour growth and progression.

Most studies investigating kinesin inhibition in lung cancer have used a limited number of common cell line or xenograft models, limiting our understanding of the generalizability of phenotypes induced and its effectiveness in heterogeneous patient-derived xenografts that more accurately model lung tumours in patients. Nevertheless, ample preclinical evidence exists to support the pursuit of several kinesins as targets in lung cancer. To date, the kinesin targets that have progressed the furthest along the translational therapeutic pathway are Eg5 and CENPE. Inhibitors of these mitotic kinesins were found to have acceptable toxicity profiles in clinical trials, with neutropenia and fatigue being the most common adverse events observed in patients [75,125]. Little neurotoxicity has been seen, which is a common, severe, and sometimes long-lasting side effect of anti-mitotic microtubule-binding agents like taxanes [75,125,238]. Unfortunately, however, besides the combinatorial activity of the Eg5 inhibitor, filanesib, with bortezomab and dexamethasone in multiple myeloma [75,239], kinesin inhibitors have yielded low or no objective response rates in advanced cancer patients. This indicates that the promising anti-tumour activity observed in preclinical models has not translated to patients in the clinic. Below, we discuss several challenges that must be addressed to drive progress in the development of effective kinesin-targeted therapies to unleash their potential to benefit lung and other cancer patients.

### 6.1. Potency and Selectivity of Kinesin Inhibitors

Established kinesin-targeted inhibitors and drugs demonstrate the feasibility of inhibiting kinesins pharmacologically. While clinical-grade inhibitors of Eg5, CENPE, and KIF18A have been reported to selectively and potently inhibit their targets [128,135,240,241], the specificity and/or potency of other kinesin inhibitors has been poor, suboptimal, or not robustly characterized. For instance, numerous KIFC1 inhibitors have been evaluated preclinically, but none have progressed to clinical studies [188]. The first KIFC1 inhibitor described, AZ82, induced non-specific cytotoxicity in yeast and exhibited poor bioavailability in rodents [188,242]. Our own work characterizing the sensitivity of LUAD models to KIFC1 loss of function revealed that AZ82 exhibited the strongest cytotoxic effects in a model with low KIFC1 expression that was insensitive to genetically mediated KIFC1 depletion [189]. Furthermore, micromolar concentrations of AZ82 and other KIFC1 inhibitors are required to achieve cytotoxic effects in cancer cells [188,189,243,244], raising the likelihood of undesirable off-target effects. These findings illustrate the need for novel inhibitors with improved selectivity and potency to harness the therapeutic potential of promising targets like KIFC1.

One obstacle limiting the development of KIFC1 inhibitors is the lack of structural knowledge needed to inform drug-binding sites and guide structure–function-driven design of compounds with improved pharmacokinetic and pharmacodynamic profiles [188]. To address this challenge, Sharma et al. recently performed an in silico drug docking analysis of available KIFC1 inhibitors to map putative drug binding sites in the KIFC1 protein, yielding structural insights that could be used to engineer novel KIFC1 inhibitors with superior pharmacologic activity [188]. Applying this conceptual framework to other kinesins that can be targeted with non-specific inhibitors (e.g., KIF2C, KIF20A, and KIF15) could also be useful. Alternatively, available compounds with documented abilities to inhibit kinesin functions could be used as scaffolds to engineer more potent and selective derivatives, and high-throughput screens could be used to discover completely new compounds. Given the success of KRAS G12C inhibitors that bind within the switch-II pocket to regulate KRAS’ affinity for GTP [245], compounds targeting allosteric sites that disrupt kinesin associations with ATP or microtubules may demonstrate enhanced kinesin-specific selectivity. Such an approach could mitigate the challenge of high homology between motor domains of different kinesins and other ATPases. We are optimistic that continued drug development efforts will guide the synthesis of small molecule inhibitors with enhanced potencies and improved kinesin specificity, ultimately resulting in clinically useful compounds.

### 6.2. Therapeutic Index

The ability of kinesin inhibitors to exert their cytotoxic effects preferentially in cancer cells will greatly influence their clinical utility. Ideal targets are kinesins whose expression is largely restricted to cancer cells and/or whose function is dispensable in non-malignant cells, as has been described for KIF20A, KIFC1, and KIF18A [98,142,145,195,196]. Observations that Eg5 and CENPE inhibitors have been well-tolerated in patients suggest that inhibiting these kinesins in normal cells is relatively innocuous [75,125]; although, on-treatment tumour biopsies would be required to confirm effective suppression of target activity. The therapeutic indices for targeting other kinesins are not as well established because their inhibition has not been robustly tested in non-malignant cells, and/or the lack of specific inhibitors has precluded their evaluation in animal models where off-tumour toxicities would be evident. As such, studies to better define the dependence of healthy, non-transformed cells on individual kinesins are imperative. At least in theory, targeting kinesins with the intent of exacerbating CIN in cancer cells with pre-existing instability widens the therapeutic window for kinesin-targeted therapy. Preclinical work demonstrating greater efficacy of CENPE, Eg5, and KIF18A inhibitors in aneuploid, tetraploid, and CIN-positive cancer models supports this concept [74,134,142,145]. This highlights the need for similar studies to determine whether other kinesins are vulnerabilities specific to cancer cells with CIN to better understand their therapeutic potential.

### 6.3. Effects of CIN in Non-Malignant Cells

It cannot be overlooked that treatments designed to potentiate CIN in cancer cells may induce low to moderate levels of instability in diploid non-malignant cells, causing cell death or rendering them susceptible to neoplastic transformation [10,12]. To address this potential adverse effect, thorough investigations of the effects of kinesin inhibition on the karyotypes of normal cells are needed. Although CENPE deletion in a model of liver regeneration in adult mice induced chromosome misalignment and CIN, livers recovered with normal hepatocyte differentiation and function [115], consistent with the idea that non-malignant cells can protect themselves from CIN and its potentially lethal consequences. Inducing genetic alterations in non-malignant cells as a byproduct of kinesin inhibition could also initiate cellular transformation, leading to secondary tumour formation [10,12]. While this unwanted effect is not trivial, it is a risk associated with many standard-of-care treatments that induce DNA damage, including chemo- and radiotherapy, especially in younger patients with longer life expectancies [246]. The development of alternative modes of delivering anti-cancer drugs and their cytotoxic effects preferentially to tumour tissues is an active area of research in oncology, exemplified by studies investigating the use of prodrugs, nanoparticles, antibody-drug conjugates, and other tumour-directed drug delivery strategies [247,248,249,250]. If effective, such approaches could be applied to kinesin-targeted therapy to increase its therapeutic index and minimize potential adverse effects caused by inducing CIN in normal cells.

### 6.4. Enhancing Efficacy with Combination Strategies

The results of trials evaluating Eg5 and CENPE inhibitors in cancer patients indicate that they are ineffective as monotherapies. Several theories to explain the failure of Eg5 and CENPE inhibitors to date have been proposed, including mitosis-specific expression of the targets combined with low mitotic indices in solid tumours, mitotic slippage to evade cell death, suboptimal pharmacodynamic and pharmacokinetic profiles, resistance conferred by mutations in the targeted kinesins or CIN-induced, and compensation by functionally redundant kinesins such as KIF15 in the case of Eg5 inhibition [113,251,252]. The most recent kinesin inhibitors to enter clinical trials are those targeting KIF18A, sovilnesib and VLS-1488. The field eagerly awaits their results, which will determine whether they also suffer from limited efficacy and tumour resistance. As noted above, Eg5 inhibition was effective when combined with bortezomab and dexamethasone in relapsed or refractory multiple myeloma patients [76]. This combination achieved an overall response rate of 39% with a median progression-free survival of 8.5 months. Therefore, combining kinesin-targeted therapy with other drugs may be useful for augmenting anti-tumour efficacy, and preclinical studies to identify rational combinations are warranted.

Several combinatorial approaches have been described. To overcome KIF15-mediated resistance to Eg5 inhibitors, combining Eg5 and KIF15 inhibitors is a logical solution. Implementing this strategy requires research to develop KIF15 inhibitors, which is currently underway [162,163]. Recently, Tucker and colleagues discovered that CENPE inhibitors could overcome resistance to microtubule-binding agents like taxanes by exacerbating CIN to induce breast cancer cell death, nominating this combination as a synergistic therapeutic approach [133]. As we have described, inhibition of many mitotic kinesins interferes with kinetochore attachments to spindle microtubules, producing unbound chromosomes that activate the SAC and induce mitotic arrest. Prolonged arrest normally induces cell death, but cancer cells can escape this via mitotic slippage, also known as checkpoint adaptation [239,253]. Thus, another rational strategy to enhance the efficacy of kinesin inhibitors is to combine them with drugs that inhibit anti-apoptotic proteins or mitotic exit [254,255,256]. Proof of concept for this approach has been shown in lung cancer models, where the Eg5 inhibitor SB743921 synergized with the BCL2L1 inhibitor WEHI-539 in two SCLC cell lines [60]. Similarly, combining filanesib with inhibition of the pro-survival factor, MCL-1, enhanced mitotic cell death in multiple myeloma cells, rationalizing studies to further explore this combination [257]. To complement these hypothesis-driven strategies, unbiased approaches could also be used to discover effective therapeutic combinations. These include high-throughput drug screens to identify compounds that synergize with kinesin inhibitors, as well as genetic screens to discover synthetic lethal interactions that could be targeted to sensitize cancer cells to kinesin-targeted therapy.

### 6.5. Biomarkers to Inform Patient Selection

As for any targeted therapy, the success of kinesin inhibitors will depend on the ability of clinicians to identify patients likely to benefit from the drugs. Therefore, studies to identify response-predictive biomarkers should also be prioritized when developing kinesin-targeted therapies. First and foremost, the expression of the target should be confirmed, requiring the establishment of robust IHC assays for measuring kinesins in tumour tissues. Second, identifying patients whose tumours exhibit CIN is essential given the therapeutic premise for kinesin-targeted therapy. Strictly speaking, classifying tumours as CIN-positive requires the detection of dynamic changes in the genome, which is not feasible with tumour biopsies that capture its state at one moment in time [8]. The expanding clinical use of liquid biopsies, which could be used to characterize DNA in circulating tumour cells, may be helpful in this regard. However, quantifying copy number alterations and aneuploidies as phenotypic indicators of CIN in diagnostic specimens is currently the most tractable approach. For this purpose, multiplexed fluorescence in situ hybridization (M-FISH) techniques can be used to detect structural and numerical chromosomal abnormalities or centromere copy numbers in tumour cells from fixed specimens [258]. Gene expression signatures of CIN, such as CIN70 [259], and next-generation sequencing assays, particularly single-cell profiling strategies that can quantify the molecular heterogeneity produced by CIN, have also been used to gauge instability [258,260]. Additional proxies for CIN include observations of abnormal chromosome segregation in mitotic tumour cells, changes in the nuclear area, and the presence of micronuclei [12]. Thus, several strategies for measuring surrogates of CIN in clinical tumour tissues exist and could be integrated into the clinic given their compatibility with different sample inputs (i.e., tissue, RNA, DNA).

Encouragingly, some CIN-related biomarkers associated with response to kinesin inhibitors have already been identified. For example, tetraploidy was associated with enhanced efficacy of Eg5 inhibitors in cancer models [74], and KIF18A inhibitors were more effective in models with whole genome duplication (WGD), aneuploidy, *TP53* mutations, and CIN [142,143,144,145]. Notably, genomic analyses of TCGA tumours indicate that a high proportion of LUAD (59%) and LUSC (64%) exhibit WGD and signatures of CIN, implicating their susceptibility to KIF18A- and Eg5-targeted drugs [13,14,15]. Additionally, our own work and the work of others have nominated centrosome amplification, a malignancy-associated phenotype with a well-established role in promoting CIN, as a putative biomarker of sensitivity to KIFC1 inhibition [189,196]. Lastly, the mitotic index of tumours may be relevant for predicting response to kinesin-targeted therapy [251,261]. As discussed above, tumours in patients often have a lower proportion of dividing cells than in preclinical cancer models. This “proliferation rate paradox” has been implicated in resistance to several anti-mitotic drugs, including those targeting mitotic kinases and kinesins, which simply may not be as effective in slow-growing cancers [251,252,261]. This suggests that biomarkers indicative of mitotic rates, such as enumeration of mitotic cells or Ki67 staining in tumour tissues, may be needed to assign kinesin-targeted therapy [262].

## 7. Conclusions

Several members of the kinesin superfamily have emerged as promising targets in lung and other tumours, providing an opportunity to develop a new class of targeted therapy for lung cancer patients. Preclinical evidence supports the concept that mitotic kinesins can be inhibited to potentiate CIN beyond a tolerable threshold in genomically unstable tumours, tipping the delicate balance of CIN and survival towards lethality (Figure 2). However, although targeting kinesins to exploit CIN is an attractive strategy, further work is needed to realize its therapeutic potential. Recently initiated clinical trials evaluating KIF18A inhibitors have reinvigorated enthusiasm for targeting kinesins in the oncology field. Insights gleaned from their results, combined with knowledge gained from unsuccessful Eg5 and CENPE inhibitors, can be leveraged to inform improved strategies for designing effective kinesin-targeted therapies. Such strategies are likely to involve novel inhibitors and alternative modes of administering them, as well as patient selection based on CIN biomarkers to enhance their therapeutic index and anti-tumour efficacy.

## Figures and Tables

**Table 1:** Kinesins relevant in lung cancer.

Family	Gene (Protein) Name	Genomic Location	Class	Type (N, M, C)	General Functions ^&^
Kinesin-1	KIF5A	12q13.3	Non-Mitotic	N, plus-end	axonal transport of vesicles, organelles, and macromolecules in neurons
	KIF5B	10p11.22	Non-Mitotic	N, plus-end	transport of organelles like mitochondria and lysosomes
Kinesin-2	KIF3A	5q31.1	Non-Mitotic	N, plus-end	cilia formation and function, intraflagellar transport, organelle transport
	KIF3C	2p23.3	Non-Mitotic	N, plus-end	microtubule dynamics, axonal growth, and regeneration
Kinesin-3	KIF1C	17p13.2	Non-Mitotic	N, plus-end	transports Golgi-derived vesicles and mRNA
	KIF13A	6p22.3	Non-Mitotic	N, plus-end	recycling endosome dynamics, intracellular cargo transport
	KIF13B	8p12	Non-Mitotic	N, plus-end	endocytosis and angiogenesis via transport of VEGFR2
	KIF14	1q32.1	Mitotic	N, plus-end	cytokinesis, chromosome congression, and alignment
	KIF16B	20p12.1	Non-Mitotic	N, plus-end	endosome transport, recycling, and degradation of receptors
Kinesin-4	KIF4A	Xq13.1	Mitotic	N, plus-end	chromosome condensation and segregation, and cytokinesis
	KIF7	15q26.1	Non-Mitotic	N, non-motile	microtubule dynamics, cilia formation, cell cycle, and Hedgehog signalling
	KIF21B	1q32.1	Non-Mitotic	N, plus-end	microtubule dynamics, cytoskeletal transport, and neuronal morphology
Kinesin-5	KIF11 (Eg5, KSP)	10q23.33	Mitotic	N, plus-end	bipolar spindle formation via centrosome separation
Kinesin-6	KIF20A (MKLP2)	5q31.2	Mitotic	N, plus-end	cytokinesis, Golgi positioning, and vesicle secretion
	KIF20B (MPP1)	10q23.31	Mitotic	N, plus-end	abscission and cytokinesis
	KIF23 (MKLP1)	15q23	Mitotic	N, plus-end	centralspindlin complex component and cytokinesis
Kinesin-7	KIF10 (CENPE)	4q24	Mitotic	N, plus-end	kinetochore-MT attachment, chromosome alignment, and spindle assembly checkpoint
Kinesin-8	KIF18A	11p14.1	Mitotic	N, plus-end	chromosome congression and alignment, and microtubule dynamics
	KIF18B	17q21.31	Mitotic	N, plus-end	chromosome alignment and microtubule dynamics
Kinesin-10	KIF22 (KID)	16p11.2	Mitotic	N, plus-end	chromosome congression, alignment, and compaction during anaphase
Kinesin-11	KIF26B	1q44	Non-Mitotic	N, non-motile	cell adhesion, migration, and polarity
Kinesin-12	KIF15	3p21.31	Mitotic	N, plus-end	spindle elongation and spindle bipolarity via centrosome separation
Kinesin-13	KIF2A	5q12.1	Mitotic	M, non-motile	spindle formation and bipolarity, chromosome alignment, and microtubule dynamics
	KIF2B	17q22	Mitotic	M, non-motile	kinetochore-MT attachment and microtubule dynamics
	KIF2C (MCAK)	1p34.1	Mitotic	M, non-motile	kinetochore-MT attachment, chromosome congression and alignment, and microtubule dynamics
	KIF24	9p13.3	Non-Mitotic	M, non-motile	microtubule dynamics and cilia formation
Kinesin-14A	KIFC1 (HSET)	6p21.32	Mitotic	C, minus-end	spindle assembly and bipolarity, and intracellular cargo transport
Kinesin-14B	KIFC3	16q21	Mitotic	C, minus-end	centrosome cohesion and spindle assembly, cytokinesis, and intracellular cargo transport

^&^ References are cited within the text; MT = microtubule.
